# Chronic Wasting Disease in Bank Voles: Characterisation of the Shortest Incubation Time Model for Prion Diseases

**DOI:** 10.1371/journal.ppat.1003219

**Published:** 2013-03-07

**Authors:** Michele Angelo Di Bari, Romolo Nonno, Joaquín Castilla, Claudia D'Agostino, Laura Pirisinu, Geraldina Riccardi, Michela Conte, Juergen Richt, Robert Kunkle, Jan Langeveld, Gabriele Vaccari, Umberto Agrimi

**Affiliations:** 1 Department of Veterinary Public Health and Food Safety, Istituto Superiore di Sanità, Rome, Italy; 2 CIC bioGUNE and IKERBASQUE, Basque Foundation for Science, Derio and Bilbao, Bizkaia, Spain; 3 National Animal Disease Center, Agricultural Research Service, United States Department of Agriculture, Ames, Iowa, United States of America; 4 Department of Infection Biology, Central Veterinary Institute of Wageningen UR, Lelystad, Netherlands; University of Alberta, Canada

## Abstract

In order to assess the susceptibility of bank voles to chronic wasting disease (CWD), we inoculated voles carrying isoleucine or methionine at codon 109 (Bv109I and Bv109M, respectively) with CWD isolates from elk, mule deer and white-tailed deer. Efficient transmission rate (100%) was observed with mean survival times ranging from 156 to 281 days post inoculation. Subsequent passages in Bv109I allowed us to isolate from all CWD sources the same vole-adapted CWD strain (Bv^109I^CWD), typified by unprecedented short incubation times of 25–28 days and survival times of ∼35 days. Neuropathological and molecular characterisation of Bv^109I^CWD showed that the classical features of mammalian prion diseases were all recapitulated in less than one month after intracerebral inoculation. Bv^109I^CWD was characterised by a mild and discrete distribution of spongiosis and relatively low levels of protease-resistant PrP^Sc^ (PrP^res^) in the same brain regions. Despite the low PrP^res^ levels and the short time lapse available for its accumulation, end-point titration revealed that brains from terminally-ill voles contained up to 10^8,4^ i.c. ID_50_ infectious units per gram. Bv^109I^CWD was efficiently replicated by protein misfolding cyclic amplification (PMCA) and the infectivity faithfully generated *in vitro*, as demonstrated by the preservation of the peculiar Bv^109I^CWD strain features on re-isolation in Bv109I. Overall, we provide evidence that the same CWD strain was isolated in Bv109I from the three-cervid species. Bv^109I^CWD showed unique characteristics of “virulence”, low PrP^res^ accumulation and high infectivity, thus providing exceptional opportunities to improve basic knowledge of the relationship between PrP^Sc^, neurodegeneration and infectivity.

## Author Summary

Chronic wasting disease (CWD) is a prion disease that affects free-ranging and captive cervids and is expanding increasingly in the USA and Canada. Animal models are of key importance in the study of prion diseases but their development for CWD has long been hampered by its very inefficient transmission to wild-type mice. Significant progress was made following the generation of transgenic mice over-expressing cervid PrP. Here we show that the bank vole (*Myodes glareolus*), a wild rodent species that we demonstrated to be susceptible to many animal and human prion diseases, is also very susceptible to CWD from elk, mule deer and white-tailed deer. Adaptation of CWD to bank vole led to the isolation of a prion strain with peculiar characteristics: unprecedented short incubation and survival times, respectively of 25–28 and ∼35 days, low PrP^Sc^ levels compared with other vole-adapted prion strains and high infectious titre. These features were all faithfully maintained upon the generation of this strain *in vitro* by protein misfolding cyclic amplification. The development of a model for prion diseases that led to disease in less than one month accumulating high infectious titres but low PrP^Sc^ levels, represents a significant tool for investigating the still unclear relationship between PrP^Sc^, neurodegeneration and infectivity in prion diseases.

## Introduction

Chronic wasting disease (CWD) of cervids belongs to the family of transmissible spongiform encephalopathies (TSE) or prion diseases, a group of fatal neurodegenerative pathologies affecting animals and humans. They are characterised by spongiform changes, gliosis and the deposition in the brain of a post-translational misfolded isoform (PrP^Sc^) of the host-encoded cellular prion protein (PrP^c^). Prion diseases also include Creutzfeldt-Jakob disease (CJD) of humans, scrapie of sheep and goats and bovine spongiform encephalopathy (BSE) of cattle.

CWD is the only prion disease known to affect free-ranging wild animals. It was first described in the United States in the late 1960s [1]. Currently, CWD has been diagnosed in farmed and free-ranging cervids in several areas of North America [2] and South Korea, where it was accidentally imported from Canada [3]. The disease has been documented in Rocky Mountain elk (*Cervus elaphus nelsoni*), mule deer (*Odocoileus hemionus*), white-tailed deer (*Odocoileus virginianus*) and moose (*Alces alces*) [4].

Epidemiological evidence indicates that CWD spreads naturally with relative efficiency and recent trend analyses suggest that its prevalence is increasing [5].

The development of laboratory models for CWD has long been hampered by the very inefficient transmission of CWD to wild-type mice [6]. Significant progress was made by the generation of transgenic mice over-expressing cervid PrP [6], [7], [8], [9], [10], [11]. CWD has also been transmitted, albeit less efficiently, to transgenic mice over-expressing mouse [12] or Syrian hamster PrP, as well as to different hamster species [13]. Recently, CWD was successfully transmitted to different species of North American wild rodents [14] and ferrets [15].

In recent years, with the aim of developing new animal models for prion diseases, we have studied the susceptibility of the bank vole (*Myodes glareolus*) to a wide range of human and animal prion diseases. Two lines of voles, one homozygous for methionine and the other for isoleucine at codon 109 of PrP – here designated Bv109M and Bv109I, respectively – were investigated. Bv109M was shown to be susceptible to sporadic and genetic CJD [16], [17], sheep scrapie [18], [19], mouse- and hamster-adapted scrapie strains [20], [21], [22], cattle and sheep BSE [16], [22] and atypical BSE [22]. Overall susceptibility of Bv109I was found to be comparable to that of the methionine-carrying line, although with differences depending on the specific prion disease (unpublished data).

In the present study, we investigated the susceptibility of bank voles to CWD. To this purpose, we inoculated Bv109M and Bv109I with various CWD sources and found that CWD replicated faster in Bv109I compared to Bv109M. We then focused on the Bv109I line, which showed unprecedented short survival times upon CWD adaptation. We describe a thorough characterization of Bv109I-adapted CWD, showing that in spite of their short survival time, high infectious titres accumulate in the brains of affected animals. Furthermore, we provide evidence that Bv109I-adapted CWD can be easily and faithfully replicated *in vitro* by serial automated PMCA (saPMCA).

## Results

### Bank voles are susceptible to CWD isolates from three cervid species

All CWD isolates transmitted with 100% attack rate and short survival times in both Bv109I and Bv109M (Table 1). In some groups survival times were not uniform. Indeed, while most of the voles inoculated were clinically affected between 150 and 350 days post-infection (d.p.i.), a minor proportion of voles showed much longer survival times (Figure S1). Interestingly, Bv109I replicated CWD faster than Bv109M, with 60–100 days shorter median survival times (Figure S1). Conversely, SS7B, a scrapie isolate previously shown to replicate efficiently in Bv109M [19], showed a 60 days longer median survival time in Bv109I. Overall, these data demonstrate that the 109 methionine/isoleucine variation influences the susceptibility of voles in a strain dependent fashion and that Bv109I is a fast model for CWD replication. We thus further investigated the transmission features of CWD in the bank vole line expressing isoleucine at codon 109.

**Table 1:** Survival times of Bv109I and Bv109M after primary transmission of CWD.

		Bv109I		Bv109M	
		Survival time (mean±SD)	No. of affected/No. of inoculated	Survival time (mean±SD)	No. of affected/No. of inoculated
Elk	**CWD1**	190±25	16/16	280±97	18/18
	**CWD2**	226±123	18/18	278±37	18/18
	**CWD4**	267±232	7/7	253±50	8/8
Mule deer	**CWD3**	281±233	7/7	273±54	7/7
White-tailed deer	**CWD5**	156±24	6/6	253±29	11/11
	**CWD7**	171±18	5/5	259±32	12/12
	**CWD8**	169±22	6/6	249±16	12/12
Sheep	**SS7B**	257±42	15/17	188±33*	14/14*

* Published in Di Bari et al., 2008.

### Transmission features of CWD isolates in Bv109I

CWD isolates transmitted in Bv109I with mean survival times ranging from 156 to 281 d.p.i. (Table 1). All isolates induced a similar clinical picture. The onset of disease was characterised by slight behavioural alterations; hypoactivity and hyporeactivity were sometimes observed along with the disappearance of the typical behaviour of hiding under the sawdust lining the cage. Dorsal kyphosis, slight ataxia of the forelimb and occasional head bobbing (upward movements of the head) accompanied the progression of the disease. Overall, clinical signs were faint compared with those previously observed in voles infected with classical scrapie [19] and occasionally animals were found dead because of the rapid progression to terminal stage. The three isolates from white-tailed deer gave the shortest survival times, ranging from 156 to 171 d.p.i., while the survival times were slightly longer with isolates from elk and mule deer. As mentioned above, individual voles showing a late clinical onset were observed in CWD2, with two outliers at 414 and 656 d.p.i., in CWD3 (outlier at 805 d.p.i.) and CWD4 (792 d.p.i.) (Figure S1).

**Figure 1:**
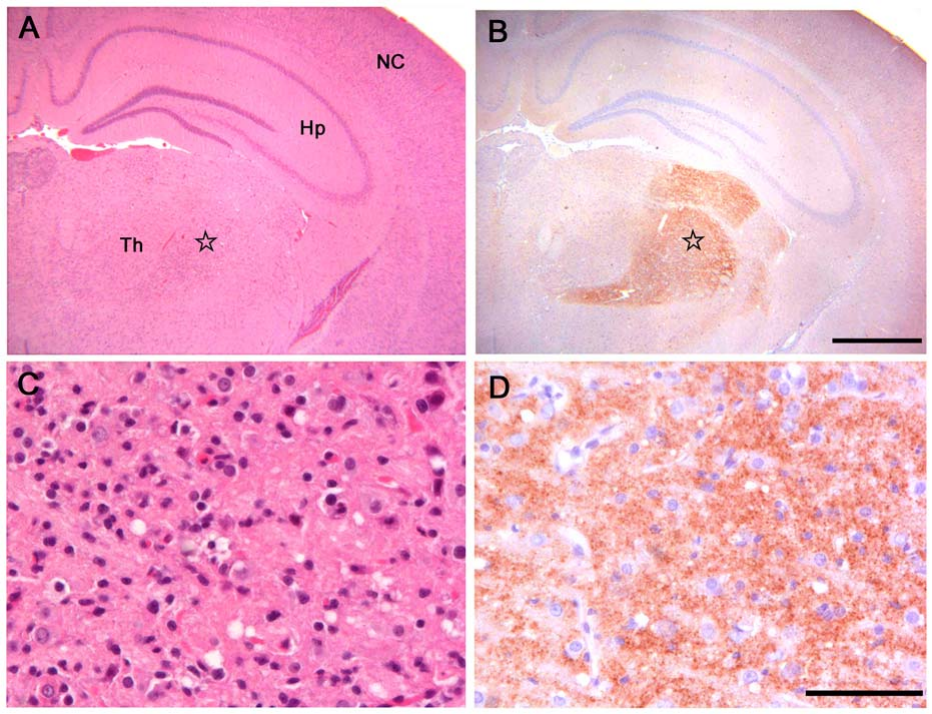
Histopathological and immunohistochemical analysis of Bv109I inoculated with Bv^109I^CWD. (A) Serial sections showing neocortex (NC), hippocampus (Hp) and thalamus (Th), stained by haematoxylin and eosin and (B) by immunohistochemistry for PrP^Sc^ with SAF84 mAb - bar 500 µm. (C) and (D): magnification of the ventral thalamic nucleus (star) from (A) and (B), respectively - bar 50 µm.

**Figure 2:**
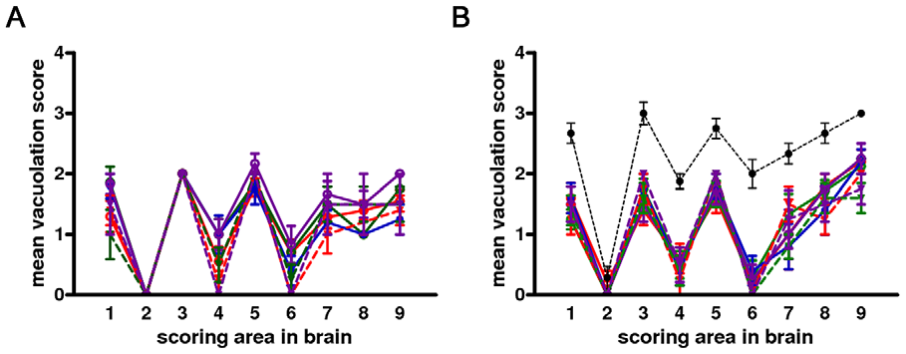
Lesion profiles of Bv109I infected with CWD following primary transmission and third passage. Pathological phenotypes observed after primary transmission and third passage are shown in (A) and (B), respectively. CWD1, 2 and 4 from elk, are shown in red, green and violet lines with open circles, respectively; CWD3 from mule deer, in blue line and closed circles; CWD5, 6 and 7 from W-T deer in red, green and violet dashed lines and closed triangles, respectively. Vole-adapted sheep scrapie is shown in black dashed line and closed circles. Brain-scoring areas are: medulla (1), cerebellum (2), superior colliculus (3), hypothalamus (4), thalamus (5), hippocampus (6), septum (7), retrosplenial and adjacent motor cortex (8), cingulate and adjacent motor cortex (9).

All infected voles showed moderate spongiform changes at brain histopathology, accompanied by gliosis and neuronal loss (Figure 1). All groups had a similar distribution of spongiform degeneration, which mainly involved the superior colliculus, the thalamus and, to a lesser extent, the cerebral cortex (Figure 2A). At the cortical level, spongiosis preferentially involved the V and VI deep layers (data not shown). Interestingly, spongiosis was not observed in the cerebral cortex of four voles surviving longer than 400 d.p.i. (Figure S2), although they showed spongiform degeneration in the mesencephalon and diencephalon with a distribution similar to that of the other infected voles (data not shown).

PrP^Sc^ was readily detected in all infected voles by IHC, PET-blot and WB. IHC showed punctate PrP^Sc^ deposition (Figure 1) restricted to specific areas. The thalamus, substantia nigra, geniculate and vestibular nuclei were the most involved areas. The hippocampus was mostly spared, though some fibres of CA2 were occasionally found positive. Overall, a topographical correlation between vacuolation, neuronal loss and PrP^Sc^ deposition was observed (Figure 1).

**Figure 3:**
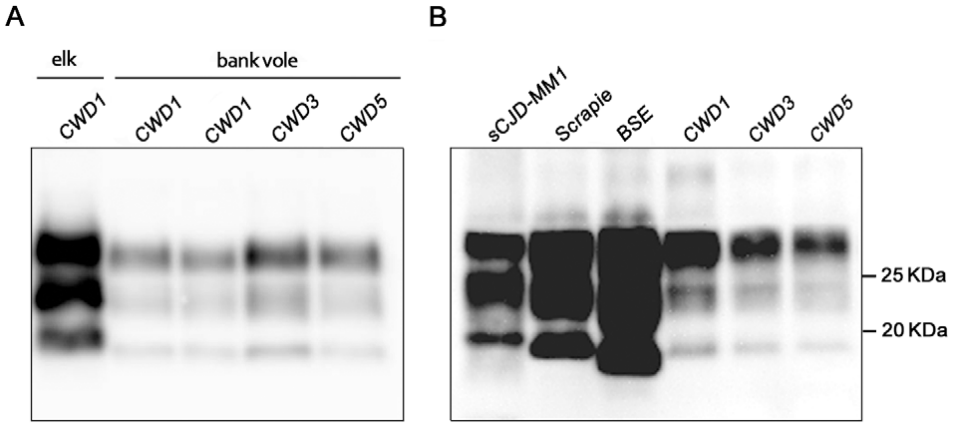
Western blot analysis of the PrP^res^ level in the brain of CWD-affected voles. (A) Immunoblot of proteinase K–resistant PrP^Sc^ (PrP^res^) from one original elk isolate (CWD1) and from voles infected with CWD1, 3 and 5. (B) Comparison of PrP^res^ amount and glycoprofile between Bv109I-adapted CWD and Bv109M-adapted sporadic CJD, sheep scrapie and cattle BSE, at third passage. Membranes were probed with SAF84. Molecular size markers are shown in kilodaltons on the right.

Western blot analysis showed the typical three-band PrP^res^ pattern, which was similar in all CWD-infected voles (Figure 3). Compared with elk and deer PrP^res^, bank vole CWD PrP^res^ showed a lower molecular mass of the unglycosylated band (Figure 3A), similar to that reported in prairie voles infected with mule deer CWD [23]. In keeping with the discrete brain distribution of PrP^Sc^ observed by PET-blot or IHC, the overall level of PrP^res^ detected in brain homogenates was much lower than that usually found in vole-adapted strains [16], [19], [22]. Direct comparison with Bv109I-adapted SS7B showed that PrP^res^ levels were at least 30 fold lower in Bv109I-adapted CWD (Figure S3). In addition, the migration pattern was different from that previously reported in bank vole TSEs, being intermediate between scrapie and BSE (Figure 3B).

All the twelve control challenged voles, culled at the end of the experiment (810 d.p.i.) or found dead for intercurrent disease (two voles, at 550 and 721 d.p.i.), were found PrP^Sc^ negative by Western blot analysis.

### CWD adapts to Bv109I with strikingly short survival times

In order to study the adaptation of CWD prions in Bv109I and to compare more closely the biological properties of the various CWD isolates, vole-passaged CWD isolates were further propagated in Bv109I. On the second passage we observed a dramatic decrease in the incubation times, with behavioural alterations already evident at 25–28 d.p.i.. Following a short clinical phase all CWD isolates produced strikingly short mean survival times of between 34 and 44 d.p.i. (Table 2). Survival times were stable or slightly shorter on third passage, with all Bv109I-adapted CWD strains giving a survival time of ∼35 d.p.i. (Table 2).

**Table 2:** Survival times of Bv109I and Bv109M after subsequent passages of CWD.

		Second passage		Third passage		Fourth passage	
		Donor (d.p.i.)	Survival time (mean±SD)	Donor (d.p.i.)	Survival time (mean±SD)	Donor (d.p.i.)	Survival time (mean±SD)
Elk	**CWD1**	181	35±3 (10)	34	37±3 (9)	34	35±2 (7)
	**CWD2**	173	35±3 (6)	35	34±2 (11)		
	**CWD4**	165	34±1 (7)	39	33±1 (6)		
		792	134, 169	134	54±5 (8)	49	33±2 (6)
						60	35±4 (6)
Mule deer	**CWD3**	167	40±1 (6)	32	32±2 (6)		
		805	164±8 (6)	176	42±4 (8)	47	34±1 (6)
White-tailed deer	**CWD5**	146	40±3 (8)	38	34±0 (6)		
	**CWD7**	148	42±3 (8)	38	33±2 (6)		
	**CWD8**	149	44±3 (7)	43	34±3 (6)		
Sheep	**SS7B**	209	73±4 (12)	71	69±6 (12)		

The number of animals in each group is reported in brackets. The survival times of voles used for vole-to-vole transmissions, indicated as ‘donors’, are shown.

**Figure 4:**
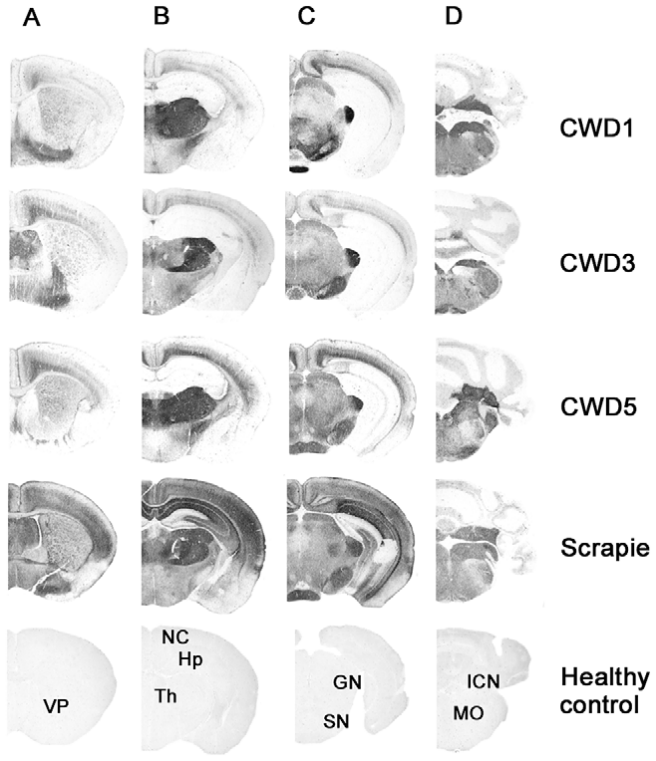
Regional distribution, by PET-blot, of PrP^res^ following the third passage of CWD1, CWD3, CWD5 and sheep scrapie in Bv109I. Coronal sections of the forebrain representing: telencephalon (A), diencephalon (B), midbrain (C) and hindbrain (D). In the lower part of the figure, the labelled coronal sections of negative control brain from 150 days old Bv109I are shown: VP, ventral pallidum; NC, neocortex; Hp, hippocampus; Th, thalamus; GN, geniculate nuclei; SN, substantia nigra; ICN, interposed cerebellar nucleus; MO, medulla oblongata.

The neuropathological phenotype after the second and subsequent passages was similar to that observed on primary transmission (Figure 2B), with lesion profiles showing a slight increase in cortical involvement (Figure 2B). The pattern of brain PrP^Sc^ distribution by PET-blot was the same in all groups, characterised by PrP^Sc^ deposition mainly in the thalamus, substantia nigra, geniculate nuclei, vestibular nuclei and the deep layers of the cerebral cortex (Figure 4). Bv109I-adapted SS7B showed a marked neurodegeneration in all areas considered in lesion profiles, with the exception of the cerebellar cortex. PET-blot showed abundant PrP^Sc^, distributed throughout the brain. Prominent immunolabelling was observed in the cortices, septal nucleus, hippocampus, thalamus, superior colliculi, geniculate nuclei and medulla oblongata.

Overall these data suggest that the same strain, designated Bv^109I^CWD, was isolated from elk, mule deer and white-tailed deer.

### Strain characterisation from Bv109I outliers suggests the isolation of more than one prion component in primary transmission

Two voles, designated CWD3^outlier^ and CWD4^outlier^, which developed clinical signs after unusually long times of 805 and 792 d.p.i. at primary transmission of CWD3 and CWD4 were selected for supplementary strain typing. Second passages from CWD3^outlier^ and CWD4^outlier^ gave much longer survival times compared with CWD3 and CWD4 (Table 2). At subsequent passages the survival time decreased progressively and CWD3^outlier^ and CWD4^outlier^ were fully adapted only at the fourth passage (Table 2). Interestingly, neuropathological assessment at the second (data not shown) and third (Figure S2) passages showed much less severe spongiform changes in the cortex and superior colliculus than Bv^109I^CWD at ∼35 d.p.i.. By the fourth passage, the lesion profiles converged to Bv^109I^CWD (Figure S2) and the survival time decreased to ∼35 d.p.i.. These data suggest that the Bv^109I^CWD strain was eventually isolated also from CWD3^outlier^ and CWD4^outlier^, although it required four subsequent passages to emerge. The deviant neuropathological profile observed in CWD3^outlier^ and CWD4^outlier^ at first passage was propagated for at least two vole-to-vole passages, suggesting that a second CWD strain was isolated in these outliers, which was progressively outcompeted by the extreme rapidity of the Bv^109I^CWD strain.

### Characterisation of Bv^109I^CWD

**Figure 5:**
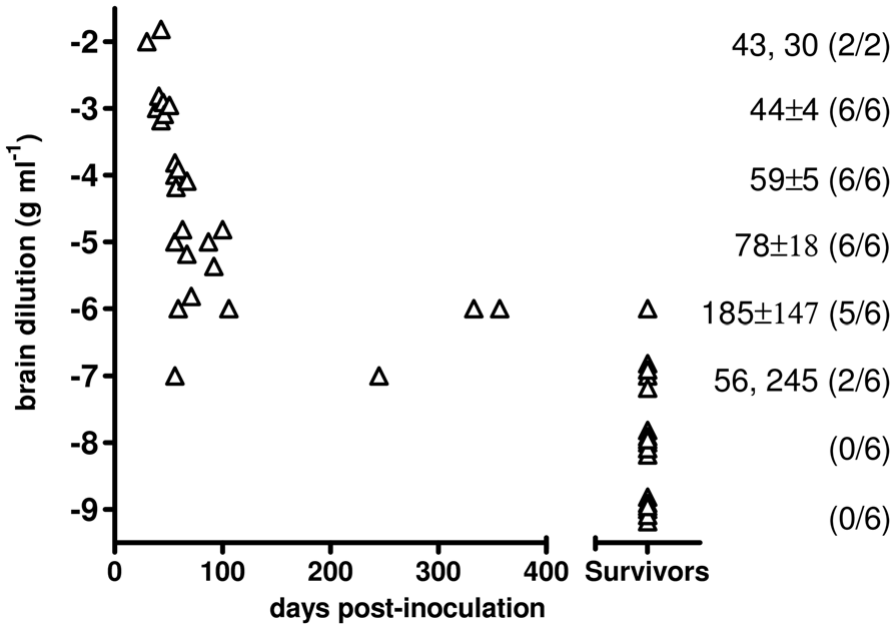
End-point titration of Bv^109I^CWD infectivity in Bv109I. Triangles represent individual survival times. Voles that did not show signs of infection after 450 d.p.i. are plotted in compressed form after the x axes break point. The mean survival times (days ± standard deviation) and the numbers of diseased/inoculated voles are indicated on the right of each chart.

The infectious titre of Bv^109I^CWD was determined by endpoint titration. Increasing survival times were observed with 10^−2^ to 10^−5^ dilutions, while dilutions higher than 10^−5^ also produced a decreasing attack rate (Figure 5). The infectious titre of Bv^109I^CWD was 10^8,4^ i.c. ID_50_ U g^−1^. The vast majority of diseased animals succumbed within ∼100 d.p.i., and only 3 out of 44 voles developed the disease later on, with unequivocal clinical signs (Figure 5). Thus Bv^109I^CWD undergoes very fast replication kinetics, allowing high prion titres to accumulate in a very short time.

**Figure 6:**
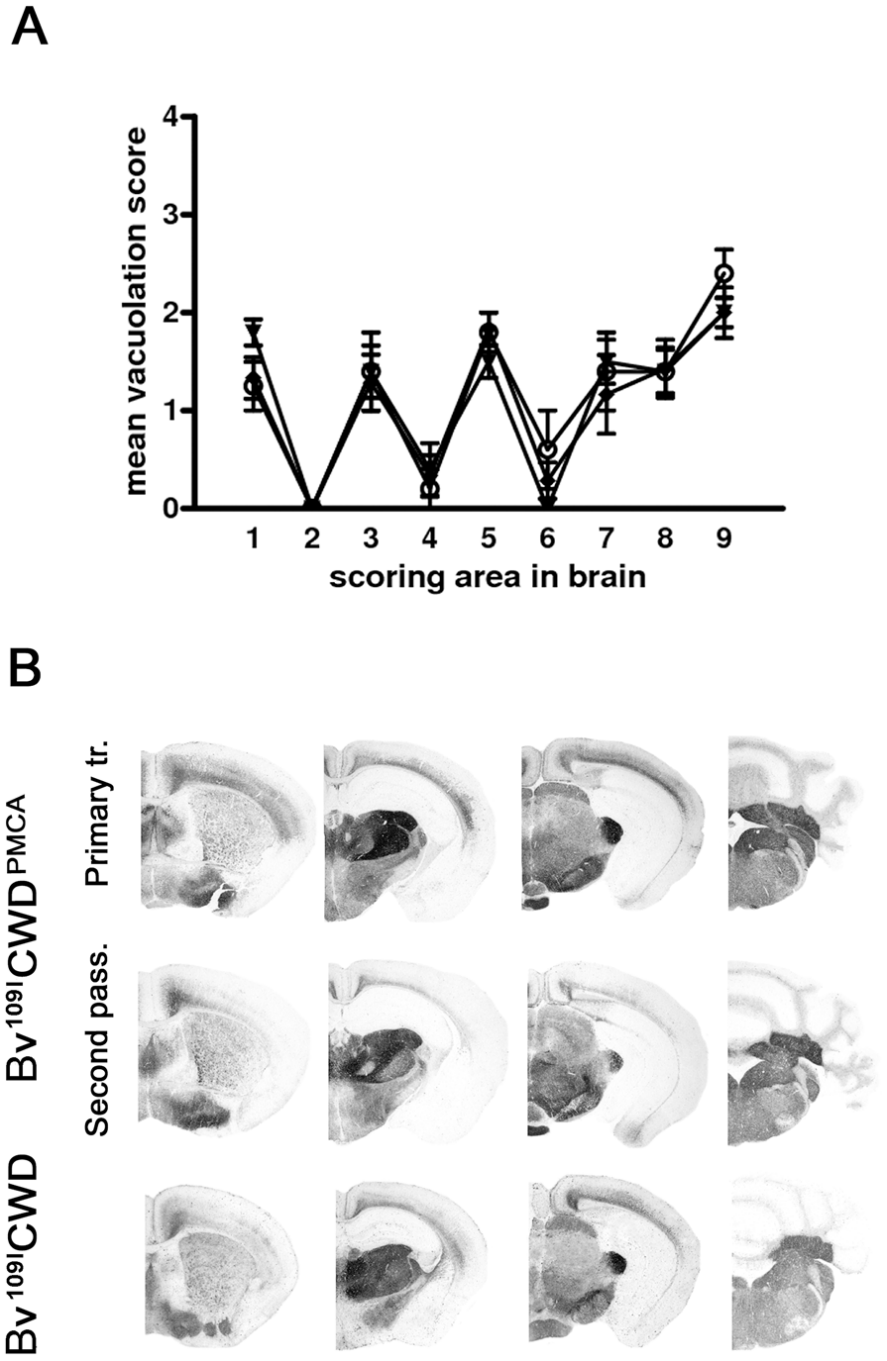
Biological properties of *in vitro*-generated Bv^109I^CWD (Bv^109I^CWD^PMCA^). A) Lesion profiles of Bv109I infected with Bv^109I^CWD^PMCA^ following primary transmission (open circles) and second passage (closed diamonds), compared with Bv109I infected with Bv^109I^CWD (triangles). Brain-scoring positions are described in Figure 2. B) Regional distribution, by PET-blot with SAF84 mAb, of PrP^res^ in Bv109I infected with Bv^109I^CWD^PMCA^ following primary transmission and second passage compared with the control group (Bv^109I^CWD). Brain areas are as in Figure 4.

To analyse the potential of Bv^109I^CWD to be amplified *in vitro*, we performed PMCA experiments using 1/100 mixtures of Bv^109I^CWD as seed and brain homogenates from Bv109I voles as substrate. As already shown with other vole species [24], Bv^109I^CWD was efficiently amplified already at the first PMCA round (amplified products from rounds 4 to 13 are shown in Figure S4). In order to verify whether Bv^109I^CWD infectivity was efficiently propagated during saPMCA and to study its biological properties in comparison with the original Bv^109I^CWD, we performed saPMCA experiments for 15 successive PMCA rounds so that the original seed was diluted 10^16^-fold. This dilution was such that only the newly generated PrP^Sc^ was theoretically present in the final PMCA product. Groups of nine voles were inoculated with 10-fold dilutions of either *in vitro*-generated Bv^109I^CWD (designated Bv^109I^CWD^PMCA^) or the original Bv^109I^CWD used as seed. Bv^109I^CWD^PMCA^ induced terminal disease in 42±2 d.p.i., a survival time similar to that of the control Bv^109I^CWD inoculum (38±2 d.p.i.). The second passage of Bv^109I^CWD^PMCA^ produced disease in 34±2 d.p.i.. The clinical phenotype and the lesion profile of animals infected with Bv^109I^CWD^PMCA^, after primary transmission and second passage, were indistinguishable from the control group (Figure 6A). The type of PrP^Sc^ deposition and the pattern of PrP^Sc^ distribution in the brain, analysed by IHC (not shown) and PET-blot (Figure 6B), were also the same in Bv^109I^CWD^PMCA^ and Bv^109I^CWD. Overall, Bv^109I^CWD produced *in vitro* was highly infectious and faithfully maintained the properties of the original Bv^109I^CWD strain.

## Discussion

In the present study we investigated the susceptibility of Bv109I and Bv109M to seven CWD isolates from three deer species and found that these animal models are highly permissive to CWD, showing 100% attack rate and mean survival times between 156 and 281 d.p.i.. A deepened transmission and characterization study of CWD was carried out in Bv109I. The susceptibility of this model appeared comparable to that of transgenic mice expressing cervid PrP [6], [7], [9], [10], [11]. The reasons for such a high susceptibility are unclear but apparently not related to a different expression of PrP^C^. As a matter of fact, its expression level in Bv109I is comparable to that of mouse and hamster (Figure S5). The dramatic drop in survival time with vole-to-vole sub-passages suggests that CWD still encounters a high transmission barrier in Bv109I, implying that Bv109I permissiveness to CWD was not due to the absence of transmission barrier, as observed in transgenic mice expressing cervid PrP. Using Bv109M voles we have previously shown that this species is permissive to a variety of human and animal prion diseases. Studies aimed at investigating the molecular basis of the susceptibility of bank voles to foreign prions and their selective strain preferences suggested that two asparagine residues at positions 150 and 170, specific to vole PrP, might play a role [18], [22], [25]. Interestingly, cervid PrP also has asparagine at residue 170, suggesting that sequence identity at codon 170 might facilitate the transmission of CWD to Bv109I. This interpretation is supported by the relative ease of transmission of CWD to meadow voles [14], which also have asparagine at positions 150 and 170, as well as by studies in MoPrP^170N, 174T^ transgenic mice [24], [26] and by *in vitro* amplification of CWD by PMCA [27].

A striking feature of Bv109I-adapted CWD was the short incubation time of less than one month. In an earlier work, we showed that bank voles and related rodent species have peculiarly short survival times after infection with adapted prions, and presumably support equally fast prion replication kinetics, possibly due to the previously mentioned 150N–170N PrP residues [22]. This was also observed *in vitro* using Bv109M brain homogenates as substrate for PMCA-driven prion replication [28]. Notwithstanding this, our previous and on-going studies with Bv109M have not shown evidence of ultra-fast strains such as Bv^109I^CWD, and the fastest strains observed so far in voles show incubation times of ∼2 months [19, unpublished data). Transmission studies of CWD to Bv109M have not yet completed but we have observed that also CWD adapts to Bv109M with survival times longer than Bv^109I^CWD (60–100 d.p.i.) (unpublished data). The short survival time of Bv^109I^CWD is also unprecedented when compared with those found in transgenic mouse models, in which PrP overexpression greatly fosters prion diseases [6], [7], [8], [9], [10], [11]. Indeed, the fastest rodent models reported so far, i.e. Tga20 [29], Tg52NSE [30], Tg7 [31], Tg4053 [32] and Tg338 mice [33], express several-fold higher PrP levels compared with wild-type mice and have incubation periods at least twice as long as Bv^109I^CWD. Interestingly, it was recently shown that TgS3581 mice overexpressing vole PrP encoding for Isoleucine at position 109, undergo spontaneous prion disease and that it adapts to the same model with mean survival time of 35 days [34]. These findings suggest that the presence of Isoleucine at position 109 of the vole PrP plays a specific role in determining the short survival time of Bv^109I^CWD

In the present work we provide evidence that all the hallmarks of TSEs were recapitulated within one month in bank vole CWD. The finding that neurodegeneration and PrP^Sc^ deposition showed a discrete brain distribution, involving specific neuronal populations such as those in the medulla and thalamus, might suggest that Bv^109I^CWD replication primarily involves the so called clinical target areas (CTAs) which, once colonised by prions, trigger the clinical signs and death of the animals [35], [36]. A recent study showed that shorter time periods were needed to initiate the clinical phase when the 127S scrapie strain primarily targeted CTAs, as in intraperitoneally-inoculated Tg338 mice, compared with intracerebrally-inoculated mice [37]. This was accompanied by comparatively low levels of PrP^res^ and infectivity in the brain of ip-inoculated mice. In Bv^109I^CWD we also observed unusually low levels of PrP^res^, compared with those observed in most of the vole-adapted prion strains (Figure 3). However, by endpoint titration we found unexpected high prion titres in Bv^109I^CWD, 10^8,4^ i.c. ID_50_ U g^−1^, similar to those usually observed in standard hamster and mouse-adapted scrapie strains, whose incubation times are 3–10 times longer than Bv^109I^CWD. This implies that Bv^109I^CWD undergoes extraordinarily fast replication kinetics in Bv109I brain.

Several observations suggest that prion infectivity and toxicity might be uncoupled [38], [39], [40] and these observations are currently incorporated in a general model of prion replication and toxicity [41]. According to this model, neurotoxicity is mediated by a lethal PrP species, PrP^L^, which is distinct from PrP^Sc^, but its formation is catalysed during the autocatalytic replication of PrP^Sc^. Neurotoxicity may require a critical PrP^L^ concentration to be reached, which would depend on the kinetics of prion propagation. The relative levels of toxicity and infectivity are governed by the ratio of the initial rate of PrP^C^ conversion (which leads to the production of PrP^L^) to the rate of its maturation into PrP^Sc^. Thus fast prion replication in Bv^109I^CWD might have triggered the production of high levels of PrP^L^ in short time periods, leading to rapid disease onset and animal death. The low levels of PrP^Sc^ and the fast replication kinetics observed in Bv^109I^CWD are consistent with this interpretation.

A recent work showed that in mice inoculated with the RML scrapie strain the concentration of PrP^C^ did not affect the overall level of prion infectious titres at terminal disease, while it was directly related to the incubation time, suggesting that the production of PrP^L^ is directly proportional to PrP^C^ concentrations [42]. Our observations with Bv^109I^CWD, i.e. the unusually short survival time and the high prion infectious titre in a model that does not overexpress PrP^C^, suggest that the kinetics of prion propagation and toxicity are governed by mechanisms that cannot be interpreted solely on the basis of the amount of available substrate (PrP^C^).

The unique and easily distinguishable features of Bv^109I^CWD prompted us to pursue its *in vitro* propagation by saPMCA. Bv^109I^CWD PrP^Sc^ was indeed easily propagated *in vitro*, which allowed us to produce Bv^109I^CWD^PMCA^ PrP^Sc^, theoretically devoid of any PrP^Sc^ formed *in vivo*. Bv^109I^CWD^PMCA^ was highly infectious and faithfully reproduced the peculiar phenotype of Bv^109I^CWD. These findings confirm that CWD prions can be generated *in vitro*, as already demonstrated by others using transgenic mice expressing cervid PrP [43], [44], [45], [46] and prairie voles [47]. Furthermore, given the unique characteristics of this strain, it is extremely unlikely that their faithful maintenance during saPMCA could have occurred by chance and our results represent a convincing confirmation of other studies that have already demonstrated the ability of PMCA to replicate prion strains faithfully [44], [45], [47], [48], [49].

Elk, mule deer and white-tailed deer are the species most affected by CWD. The homogeneous and peculiar phenotypes observed in Bv109I inoculated with CWD isolates from these three cervid species indicate that the same CWD strain was isolated from all species. Interestingly, Bv^109I^CWD was isolated not only from natural cases of disease in elk (CWD1, 2 and 4) and mule deer (CWD3), but also from white-tailed deer experimentally inoculated with CWD-affected white-tailed deer, mule deer and elk (CWD5, 7 and 8, respectively). Along with previous findings in transgenic mice expressing cervid PrP [6], [9], and in keeping with the ease of indirect horizontal transmission of CWD [50], these data suggest that the same CWD strain circulates among different cervid species and maintains its characteristics following interspecies transmission. Recently, a large transmission study with elk and mule deer isolates provided substantial evidence for two prevalent CWD prion strains and suggested that individual CWD inocula might contain mixtures of the two prion strains [11]. Interestingly, we found similar evidence in at least two of the seven inocula investigated, derived from elk and mule deer, although we were unable to stabilize two different Bv109I-adapted CWD strains. Indeed, on primary transmission of CWD2, CWD3 and CWD4 inocula we observed voles that developed clinical signs after unusually long times, showing a slightly different neuropathological profile from that of voles with shorter survival times. A sub-passage in Bv109I of two of these outliers induced a survival time of 150–160 d.p.i. on second passage, compared with 35–45 d.p.i. observed with all other sub-passages. Such a long survival time might have depended on a low infectious titre in the brain from outlier voles, although they showed levels of PrP^res^ similar to the other voles of their groups. However this hypothesis is excluded when these results are compared with the survival times observed in the endpoint titration experiment, which showed that even 10^−5^ dilution of Bv^109I^CWD had a mean survival time <80 d.p.i.. In addition, the slightly deviant lesion profile observed in outlier voles was preserved for 2 sub-passages, before both the survival time and the neuropathological profile converged with the fast Bv^109I^CWD strain. Overall, our findings strongly suggest that a second CWD strain was propagated in vole outliers, which was progressively outcompeted by the extremely rapid Bv^109I^CWD strain. The “evanescent” presence of a second strain might be also interpreted, according to the quasi species nature proposed for prions [51], as the progressive elimination of the less fitting conformers occurred following interspecies transmission.

Here we demonstrate the high susceptibility of Bv109I to CWD, which adds Bv109I to the portfolio of animal models useful for the study of CWD strains. The unique properties of Bv^109I^CWD provide exceptional opportunities to improve basic knowledge of the relationship between PrP^Sc^, neurodegeneration and infectivity. The short survival time of Bv^109I^CWD, coupled with its high infectious titre, offers useful advantages for titration studies, while its unique clinico-pathological phenotype makes Bv^109I^CWD one of the best options for studies aimed at investigating strain fidelity in experimental conditions.

## Materials and Methods

### Ethics statement

Bv109I and Bv109M were obtained from the breeding colony at the Istituto Superiore di Sanità (ISS). The research protocol, approved by the Service for Biotechnology and Animal Welfare of the ISS and authorised by the Italian Ministry of Health, adhered to the guidelines contained in the Italian Legislative Decree 116/92, which transposed the European Directive 86/609/EEC on Laboratory Animal Protection.

### Samples and transmission experiments

Brain tissues from CWD affected elk (n = 3), mule deer (n = 1) and white-tailed deer (n = 3) were used for primary transmissions. Two isolates from elk, CWD1 and CWD2, were provided by the United States Department of Agriculture (Dr. A.L. Jenny) and carried the wild type PRNP genotype. The third elk isolate, CWD4, was heterozygous methionine/leucine at codon 132. It was provided by the University of Wyoming (Dr. J.E. Jewel) as the mule deer isolate, CWD3, which was homozygous for serine at codon 225. The three isolates from white-tailed deer derived from an intracerebral experimental challenge [52]. All deer were heterozygous glycine/serine at codon 96 and were challenged with a white-tailed deer CWD isolate (CWD5, code 654 in the original paper), a mule deer CWD (CWD7, code 643) or a elk CWD (CWD8, code 677). Brain sample from a healthy elk was also inoculated as negative control.

An Italian scrapie isolate from Sarda sheep (SS7B) carrying the ARQ/ARQ genotype previously characterised into Bv109M [19], was used for comparison.

Tissues were homogenised at 10% (wt/vol) in phosphate buffered saline (PBS) and stored at −80°C. The PrP^res^ amount estimated by Western blot was comparable in all the homogenates (data not shown).

Groups of eight-week-old Bv109I and Bv109M were inoculated intracerebrally with 20 µ of homogenate into the left cerebral hemisphere, under ketamine anaesthesia (ketamine 0.1 µg/g). The animals were examined twice a week until neurological signs appeared, after which they were examined daily. Diseased animals were culled with carbon dioxide at the terminal stage of the disease, but before neurological impairment was such as to compromise their welfare, in particular, their ability to drink and feed adequately. Survival time was calculated as the interval between inoculation and culling or death. After that, the brain was cut parasagittally into two parts. The smaller portion was stored at −80°C and the larger one was fixed in formalin.

The inocula for the second and third passages were prepared, as 10% wt/vol homogenates in PBS, using the brain of one of the first animals of each group that developed the disease. Supplementary second and subsequent passages from two voles that showed much longer survival times compared with the rest of their groups (outliers) were also performed.

### Titration of Bv^109I^CWD

The specific infectivity of Bv^109I^CWD was assessed in Bv109I by end-point titration using tenfold dilutions of brain homogenates from the third passage of CWD1 and calculated as ID_50_ U g^−1^ according to the Spearman and Kärber method [53]. Survivors were animals that survived until the end of the experiment (450 d.p.i.) with no sign of infection, as assessed by Western blot. The transmission rate was calculated as the ratio between voles confirmed positive by Western blot and the number of voles inoculated, excluding animals culled for intercurrent disease before 30 d.p.i..

### Histopathology

Histology, immunohistochemistry (IHC) and PET-blot analysis were performed on formalin-fixed tissues as previously described [16], [19]. Briefly, brains were trimmed at standard coronal levels, embedded in paraffin wax and cut at 6 µm for haematoxylin and eosin staining, immunohistochemistry and PET-blot. Sections were randomly mixed and coded for blind pathological assessment. For the construction of lesion profiles, vacuolar changes were scored in nine grey-matter areas of the brain on haematoxylin and eosin stained sections [16], [54], [55]. Vacuolation scores were derived from the examination of at least six voles per group. PET-blot and IHC were performed as described in Di Bari et al. [19], [26] using the SAF84 mAb.

### Western blot

CWD inocula (10% w/v homogenates in PBS) were added to PBS/sarcosyl up to a final 2% sarcosyl (Sigma) concentration and incubated for 20 min at room temperature before digestion with proteinase K (200 µg/ml) for 1 hour at 37°C with gentle shaking. Protease treatment was stopped with 3 mM PMSF.

Brain homogenates (10% w/v) from individual voles were prepared in 100 mM Tris-HCl, pH 7.4, containing 2% sarcosyl, incubated for 20 min at room temperature and then digested with proteinase K (50 µg/ml) for 1 hour at 37°C with gentle shaking. Protease treatment was stopped with 3 mM PMSF.

Electrophoresis and Western blotting were performed as previously described [16]. Briefly, samples were denatured by adding NuPage LDS Sample Buffer (Invitrogen, Carlsbad, California, United States) and NuPage Sample Reducing Agent (Invitrogen), and heated for 10 min at 90°C. After centrifugation at 10,000 *g* for 5 min each sample was loaded onto 12% bis-Tris polyacrylamide gels (Invitrogen). After electrophoresis and Western blotting on PVDF membranes (Immobilon-P; Millipore, Bedford, MA, USA), the blots were processed by SNAP i.d. Protein Detection System (Millipore) in accordance with the manufacturer's instructions. Vole PrP^res^ was detected using monoclonal antibodies SAF84 (1.2 µg/ml; epitope at amino acids 163–169 of the bank vole PrP sequence) and 12B2 (2.4 µg/ml; epitope at amino acids 89–93 of the sheep PrP sequence). Horseradish peroxidase-conjugated anti-mouse immunoglobulin (Pierce Biotechnology, Rockford, Illinois, United States) was used as secondary antibody (1∶13,000). The membranes were developed using an enhanced chemiluminescence method (SuperSignal Femto, Pierce). The chemiluminescence signal was detected using the VersaDoc imaging system (Bio-Rad) and was quantified by QuantityOne software (Bio-Rad).

### Serial automated protein misfolding cyclic amplification

A vole brain from the third passage of CWD1 was homogenised at 10% w/v in PBS and divided into two aliquots. The first aliquot was used as a seed for saPMCA while the second was used for bioassays. Substrates from Bv109I voles were prepared as 10% brain homogenates in conversion buffer (PBS, 0.15 M NaCl and 1% Triton X) and saPMCA was performed as previously described [28] by diluting 1∶100 the seed into Bv109I substrate followed by one round of PMCA using a Misonix 3000 Sonicator, with the following settings: 20 seconds of sonication every 30 minutes for 24 hours (48 cycles of incubation/sonication); 200–250 watts (potency 8) sonication power. For successive rounds of saPMCA, the product of the previous round was diluted 1∶10 into fresh substrate and subjected to a further PMCA round. This procedure was repeated 14 times to reach a 10^−15^ final dilution of the initial CWD infected brain homogenate. The detailed protocol for saPMCA has been published elsewhere [56], [57], [58]. After each round, PrP^res^ was evaluated by Western blot (Figure S4). Given the risk of contamination that is intrinsic for an ultrasensitive technique as PMCA [28], several healthy Bv109I brain homogenates were processed, as negative controls, in each round. Before each successive round, the products of the previous round were analysed by Western blot to confirm the amplification of Bv^109I^CWD and the negativity of controls.

## Supporting Information

Figure S1 **Survival times of Bv109I and Bv109M following primary transmission of CWD and sheep scrapie.** Outliers are visible among Bv109I inoculated with CWD2, 3 and 4; one outlier is visible among Bv109M with CWD1. Symbols represent individual survival times. Bars indicate the median for each group.
(TIF)

Figure S2 **Lesion profiles of Bv109I inoculated with outliers from CWD3 and CWD4.** A) Lesion profiles of Bv109I inoculated with the third passage of CWD3^outlier^ (red line and open circles) and CWD4^outlier^ (green line and closed triangles). B) Lesion profiles of Bv109I inoculated with the fourth passage of CWD3^outlier^ (red line and open circles) and CWD4^outlier^ (as shown in Table 2, two fourth passages were performed out from voles culled at 49 and 60 d.p.i. in the third passage – identified with green and green dashed lines respectively, and closed triangles) in comparison with Bv^109I^CWD (black line and closed circles). Brain areas are as in Figure 4.
(TIF)

Figure S3 **Comparison of PrP^res^ amount in Bv109I inoculated with scrapie and Bv^109I^CWD.** The amount of PrPres in Bv109I inoculated with Bv109I-adapted sheep scrapie (SS7B) and with Bv^109I^CWD was estimated by direct comparison of brain homogenate dilutions. The homogenates were treated with 50 µg/ml PK. Each sample was diluted in loading buffer after the denaturation step. The original samples (1∶1) were loaded as 0.5 mg of equivalent brain tissue. Membrane was probed with SAF84. Molecular weight markers are shown on the right.
(TIF)

Figure S4 ***In vitro*** **amplification of Bv^109I^CWD.** Western blot of Bv^109I^CWD following saPMCA. Bv^109I^CWD was diluted 10-fold into healthy Bv109I brain homogenate and submitted to a round (48 cycles) of PMCA. The amplified material was diluted 10-fold into healthy brain homogenate repeating this procedure to reach a 10^−13^ dilution of Bv^109I^CWD. Amplified samples from rounds 4 to13 were digested with 80 µg/ml of proteinase K and analysed by Western blot using monoclonal antibody D18. Control: Normal Bv109I brain homogenate.
(TIF)

Figure S5 **Comparison of PrP^C^ levels in the brain of voles, mouse and hamster.** The amount of PrP^C^ in the brain of Bv109M, Bv109I, RIII mice and hamster was assessed by direct comparison of brain homogenate dilutions (1∶1, 1∶2, 1∶4). Brain homogenates were loaded as 0.1 mg (1∶1), 0.05 mg (1∶2) and 0.025 mg (1∶4) tissue equivalents. Membrane was probed with 12B2.
(TIF)
