# Supplementary figures and images for: Chronic Wasting Disease in Bank Voles: Characterisation of the Shortest Incubation Time Model for Prion Diseases
Source: PLoS Pathog. 2013 Mar 7;9(3):e1003219. doi: 10.1371/journal.ppat.1003219 (PMC3591354; doi:10.1371/journal.ppat.1003219)

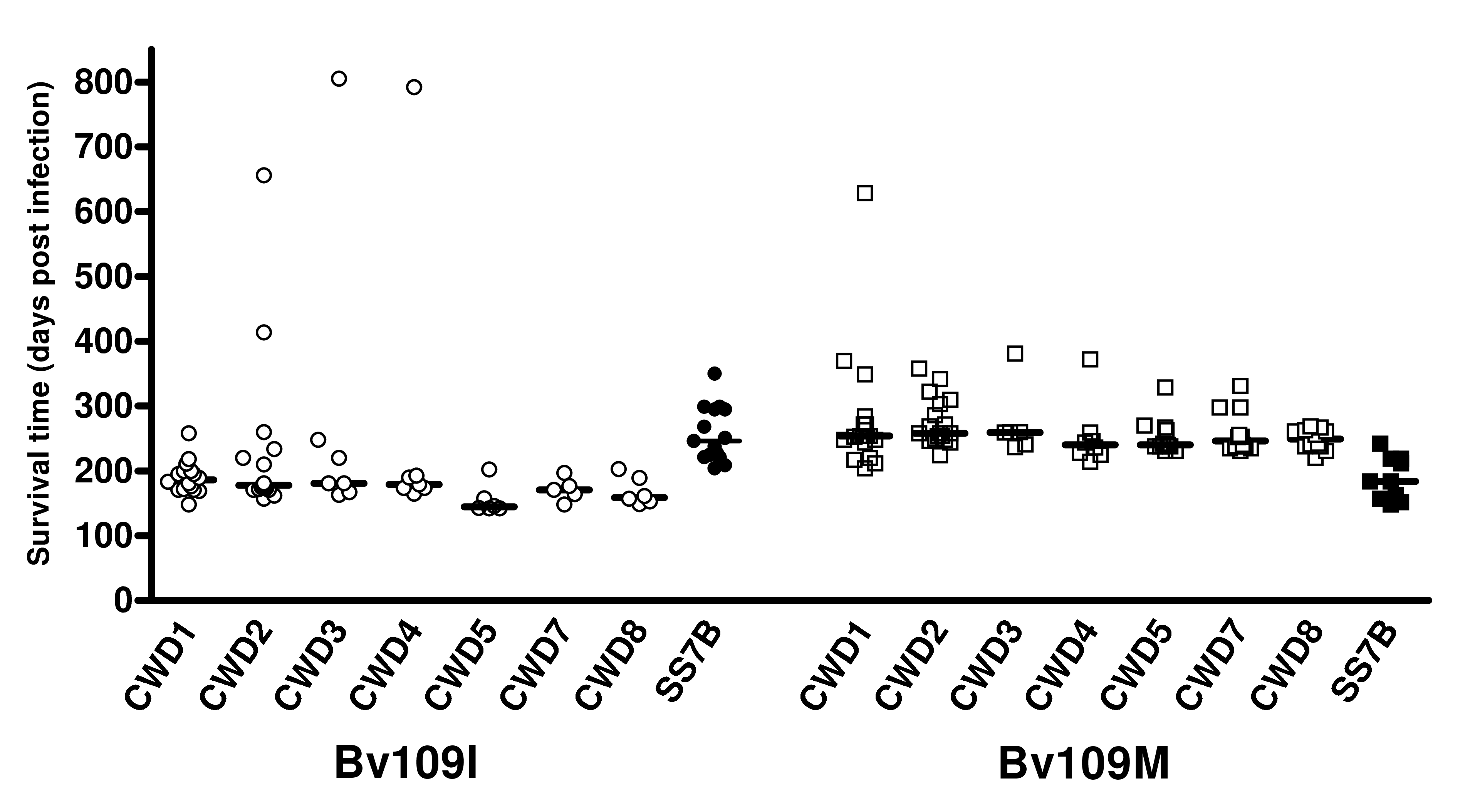

Supplement: Figure S1 — Survival times of Bv109I and Bv109M following primary transmission of CWD and sheep scrapie. Outliers are visible among Bv109I inoculated with CWD2, 3 and 4; one outlier is visible among Bv109M with CWD1. Symbols represent individual survival times. Bars indicate the median for each group. (TIF) [file ppat.1003219.s001.tif]

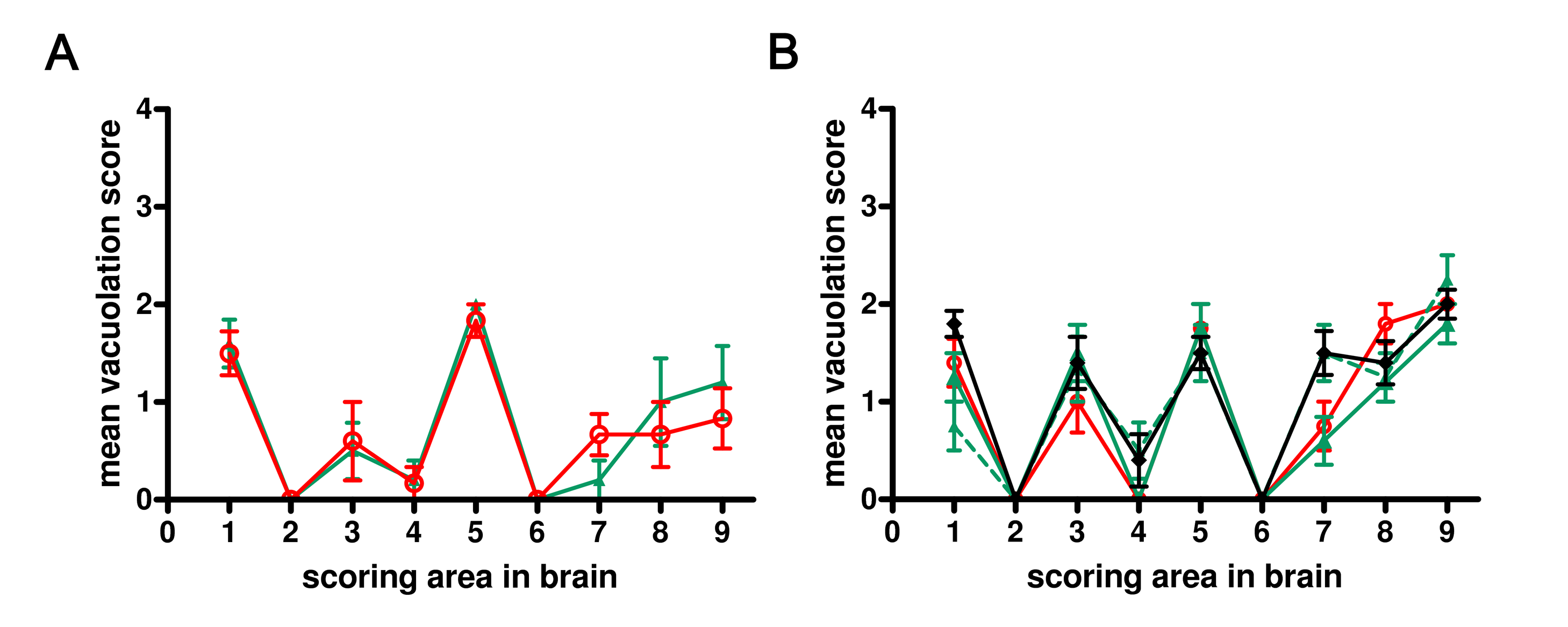

Supplement: Figure S2 — Lesion profiles of Bv109I inoculated with outliers from CWD3 and CWD4. A) Lesion profiles of Bv109I inoculated with the third passage of CWD3outlier (red line and open circles) and CWD4outlier (green line and closed triangles). B) Lesion profiles of Bv109I inoculated with the fourth passage of CWD3outlier (red line and open circles) and CWD4outlier (as shown in Table 2, two fourth passages were performed out from voles culled at 49 and 60 d.p.i. in the third passage – identified with green and green dashed lines respectively, and closed triangles) in comparison with Bv109ICWD (black line and closed circles). Brain areas are as in Figure 4. (TIF) [file ppat.1003219.s002.tif]

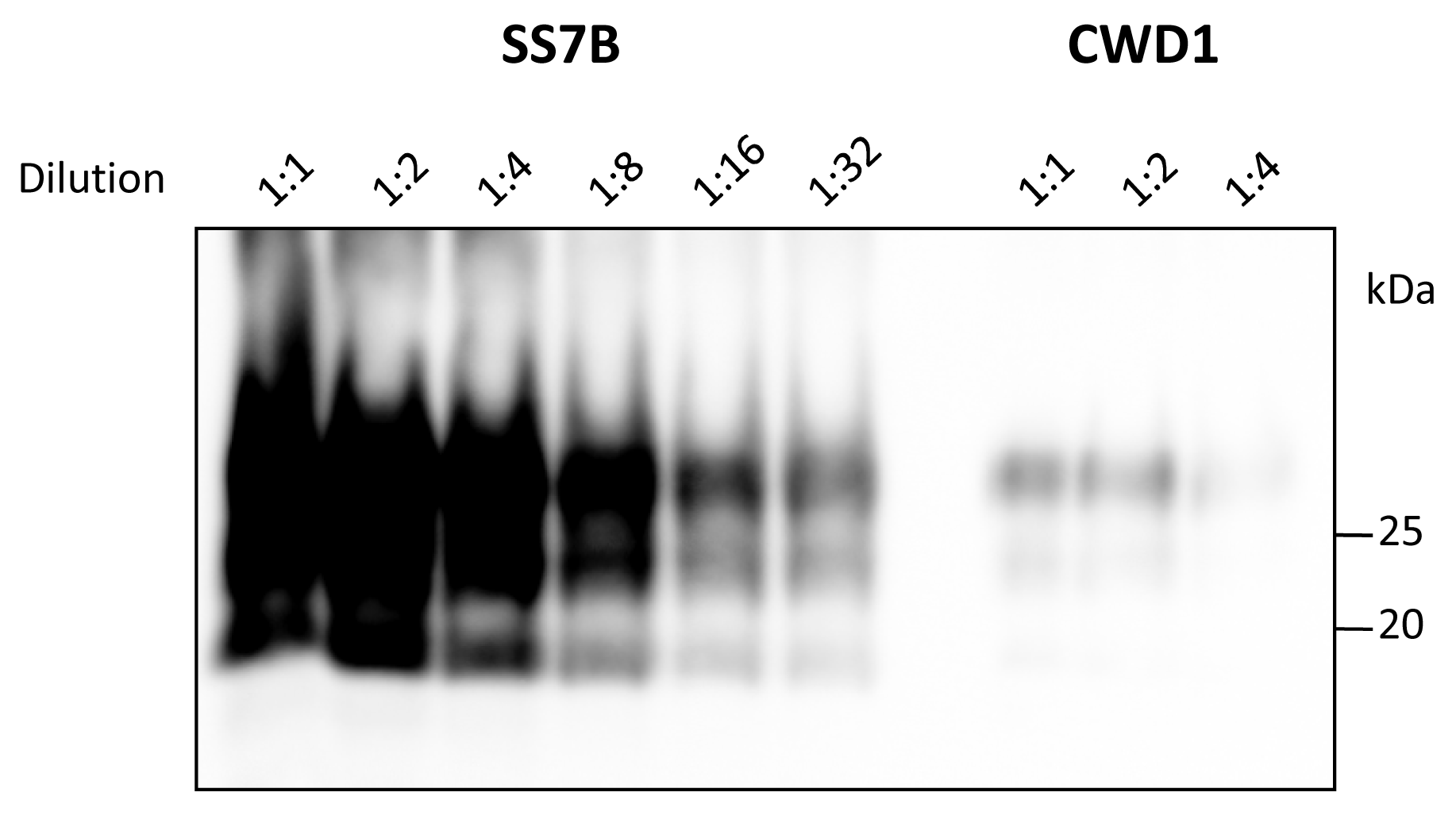

Supplement: Figure S3 — Comparison of PrPres amount in Bv109I inoculated with scrapie and Bv109ICWD. The amount of PrPres in Bv109I inoculated with Bv109I-adapted sheep scrapie (SS7B) and with Bv109ICWD was estimated by direct comparison of brain homogenate dilutions. The homogenates were treated with 50 µg/ml PK. Each sample was diluted in loading buffer after the denaturation step. The original samples (1∶1) were loaded as 0.5 mg of equivalent brain tissue. Membrane was probed with SAF84. Molecular weight markers are shown on the right. (TIF) [file ppat.1003219.s003.tif]

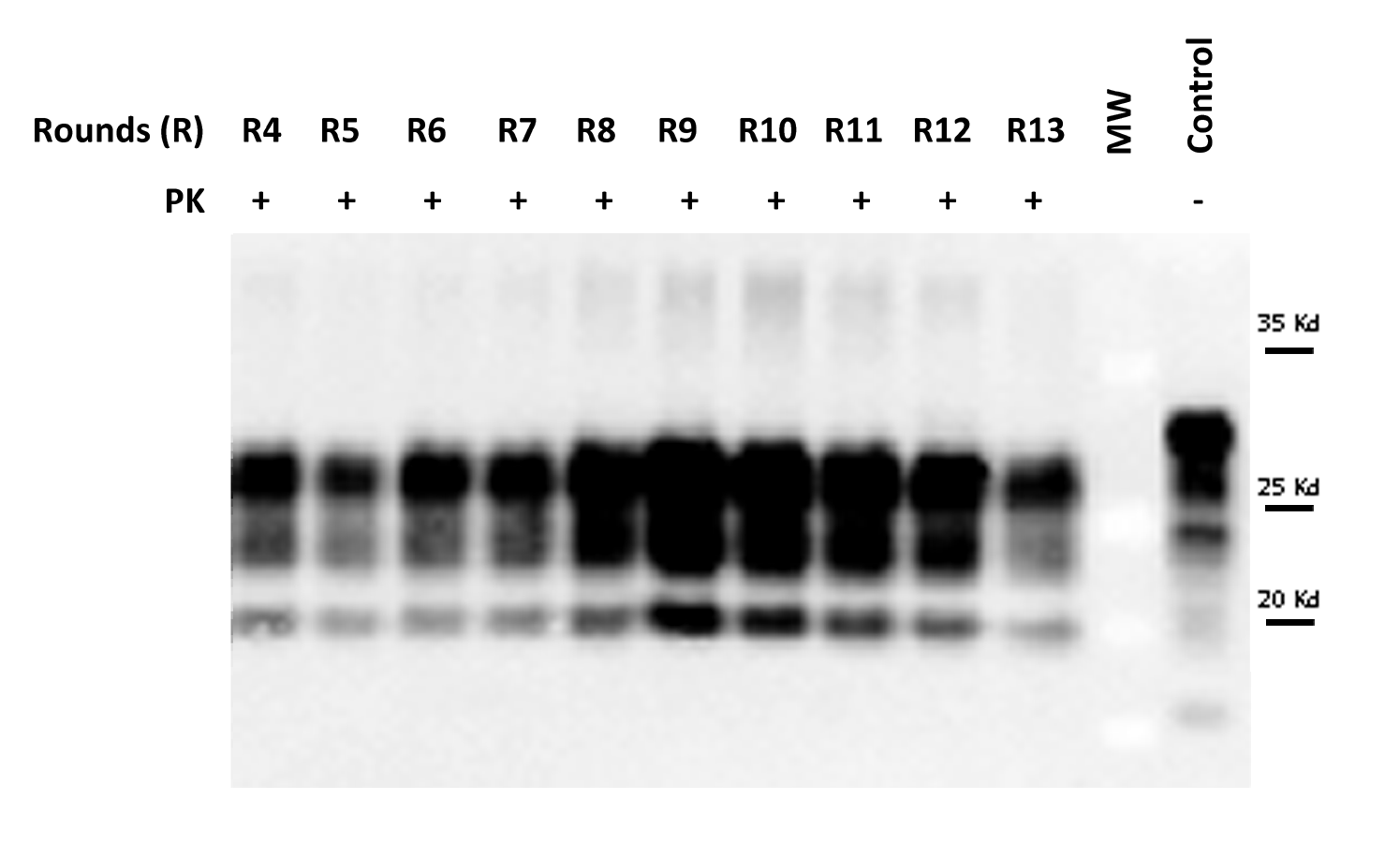

Supplement: Figure S4 — In vitro amplification of Bv109ICWD. Western blot of Bv109ICWD following saPMCA. Bv109ICWD was diluted 10-fold into healthy Bv109I brain homogenate and submitted to a round (48 cycles) of PMCA. The amplified material was diluted 10-fold into healthy brain homogenate repeating this procedure to reach a 10−13 dilution of Bv109ICWD. Amplified samples from rounds 4 to13 were digested with 80 µg/ml of proteinase K and analysed by Western blot using monoclonal antibody D18. Control: Normal Bv109I brain homogenate. (TIF) [file ppat.1003219.s004.tif]

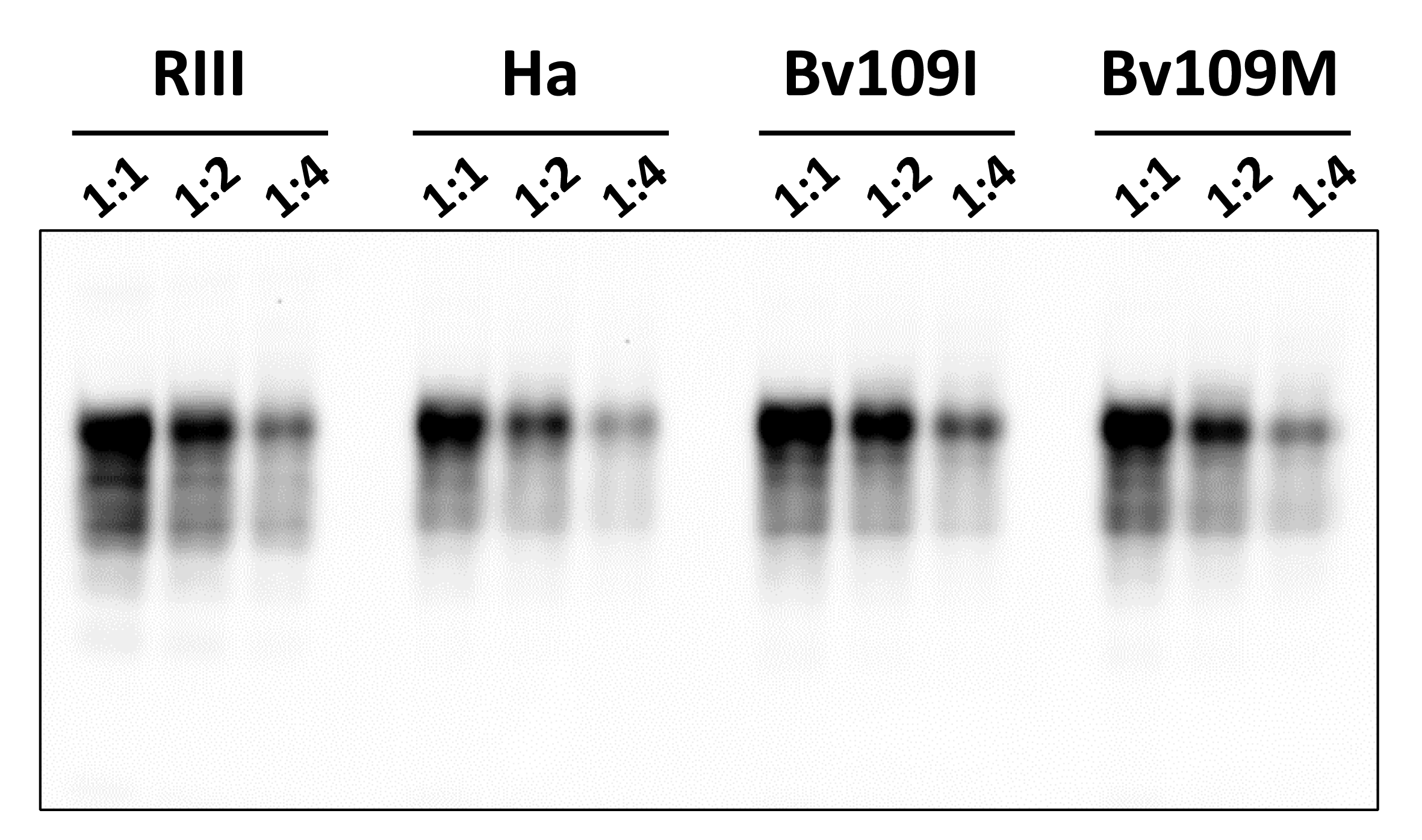

Supplement: Figure S5 — Comparison of PrPC levels in the brain of voles, mouse and hamster. The amount of PrPC in the brain of Bv109M, Bv109I, RIII mice and hamster was assessed by direct comparison of brain homogenate dilutions (1∶1, 1∶2, 1∶4). Brain homogenates were loaded as 0.1 mg (1∶1), 0.05 mg (1∶2) and 0.025 mg (1∶4) tissue equivalents. Membrane was probed with 12B2. (TIF) [file ppat.1003219.s005.tif]
